# An Audit of General Surgery Clinical Records (2021) in Ribat University Hospital, Sudan

**DOI:** 10.7759/cureus.72755

**Published:** 2024-10-31

**Authors:** Hala Fathi EmamElkhir Omer, Malaz Abusefian Elbagir Omer, Faris Salaheldin Mohamed Omer, Maab Mohammed Zain Mohammed Ali Elbashir, Sondos Omer Osman Babiker, Abeer Hussien Musa Mohamed, Hala Omer Saeed Ahmed, Marafi Mohammed Mustafa Khalil, Omaima Abdalla Hajahmed Mohamed

**Affiliations:** 1 General Surgery, Ribat University Hospital, Khartoum, SDN

**Keywords:** checklist, documentation, files, medical audit, medical records

## Abstract

Background: Documentation is critical for effective patient management in hospitals, serving essential roles in improving patient care continuity, supporting clinical decisions, and fulfilling legal requirements. Comprehensive documentation not only aids in communication among healthcare providers but also serves as a vital record of patient history, facilitating accurate diagnosis and treatment. Clinical audits are systematic evaluations that compare current patient care practices against established criteria, helping identify deficiencies and promote adherence to quality standards. By increasing awareness of documentation practices, such audits can elevate the overall standard of clinical records, leading to improved patient care and safety. This is particularly important for healthcare professionals, as accurate records are essential for licensing and certification, as well as for demonstrating the delivery of quality care.

Purpose: This study aimed to conduct a medical audit of inpatient medical records in the General Surgery Department at Ribat University Hospital to assess the documentation quality and improve patient outcomes.

Methods: A cross-sectional study was performed on 518 long-stay medical records from Ribat University Hospital in 2021. A quantitative approach was used, employing a structured checklist of 26 points as the audit tool.

Results: Documentation was largely incomplete, with significant deficiencies identified: patient full name (17.6%), admission policy (21%), admission time (2%), treatment plan approval (2%), and discharge summary (4.4%). Better documentation was found for admission dates (86.3%), medical histories (81.5%), and diagnoses (87%).

Conclusion: Accurate and comprehensive medical record documentation is essential for quality care. This audit revealed major areas needing improvement in the General Surgery Department, emphasizing the need for initiatives to enhance documentation practices.

## Introduction

Documentation is central to the management of patients in hospitals, and it is essential for research, patient care continuity, and legal defense [1] and facilitates diagnosis and treatment [2]. It also serves as a channel of communication for nurses and other medical experts, facilitating knowledge of patients' current health state [3]. It involves abstracting data from the patient file and reviewing the care given to the patient in order to determine the standard of care. One of the core components of surgical practice is the creation of operational notes. It must contain the documentation of patient information on the procedure, any further steps, and any difficulties required. For postoperative treatment, research, academic objectives, and medical-legal clarity, accurate documentation is essential. Despite the existence of guidelines instructing surgeons on how to write operating notes, shortcomings are reported everywhere. Since they are the only official record of the surgery performed on a patient and are viewed as required components of medical records, operational notes, in particular, play an essential role in patients' care [4].

A clinical audit is a quality control technique that is described as a systematic procedure to examine patient care in comparison to predetermined and accepted criteria in order to pinpoint practice gaps [5]. The audit raises awareness of the necessity of improving practices, which raises the standard of clinical records [6]. It led to enhanced administration and patient care, as well as improved systems [7]. In addition to being necessary for a healthcare professional to meet license and certification criteria, keeping an accurate record is essential for proving that a patient received quality care [8].

A doctor’s responsibility to care for patients includes keeping medical records on a regular basis. For the care of patients in general practice and for hospital documentation, forms, whether recorded on paper or in electronic format, are crucial. In addition to analyzing the medical records and data, it evaluates the accuracy of the documentation of patient records, history forms, and record summaries [9]. The majority of documentation in teaching hospitals is completed by interns, which could lead to issues because there is not enough supervision of the assistant’s performance by the attending physician [10].

Literature has demonstrated that developing countries’ health information is inaccurately registered and poorly documented [11]. The level of knowledge in Khartoum’s hospitals was good, but when compared to developing countries, the quantity and quality of their documents were lacking [12]. The lack of policy standards, procedures/guidelines, training, supervision, and auditing appears to be the cause of this documentation’s poor quality [13].

Here, at Ribat Hospital, we have taken the first step in auditing and evaluating long-stay files, which are written records that hospitals use to monitor their patients and promote communication among medical staff. Information on the patient’s identification, medical examination results, diagnosis, treatment, aftercare, and surgical notation should all be provided.

This study aims to examine the documentation process to improve documentation completeness and quality.

## Materials and methods

This observational, descriptive, cross-sectional hospital-based study was conducted in the General Surgical Department of Ribat University Hospital to assess long-stay files, conducted from November 1 to December 30, 2021.

Audit area/population

This study was conducted in the General Surgical Department of Ribat University Hospital, located in Khartoum State, Sudan. The department is part of a residency program organized by the Federal Ministry of Health and consists of six units. Each unit operates throughout the week and includes one consultant or specialist, registrars, medical officers, and house officers. The average number of registrars, medical officers, and house officers per department is four, four, and seven, respectively. The bed capacity of the Surgery Department is around 100 beds, with an average of 75 admitted patients per month.

Inclusion criteria

All long-stay files from the General Surgery Department in 2021 were included in the audit.

Exclusion criteria

Records from other surgical specialties (e.g., orthopedic, neurosurgery, cardiac) and non-surgical specialties (e.g., internal medicine, pediatrics) were excluded to concentrate specifically on general surgery.

Sample technique and sample size

All long-stay files from 2021 were collected. A total of 518 long-stay files in the General Surgery Department constituted the sample size. Long-stay files refer to any case admitted to the hospital (excluding cases admitted solely to the ER) and kept under hospital care for an extended period. These cases are considered long-stay, and their corresponding medical records are categorized as long-stay files.

Data collection method and analysis

Data collection utilized a checklist designed by the quality and excellence hospital team (Table 1), employing the iAuditor app for data collection and Microsoft Excel 2016 for data analysis.

Audit cycles

One cycle: It aimed to assess all files of the General Surgery Department for further evaluation and improvement.

Audit approval

This audit was approved by the total quality and excellence center of Al-Ribat University Hospital, serial number (RCQE20,0025), and there is no need for the patient's informed consent for this audit.

Standards

The standards are local standards developed by the Ribat Center for Quality and Excellence, and they include completeness of the following records: demographics (age, sex, patient number), medical history, physical examination, investigations, diagnosis, treatment plan, attending physician (consultant, registrar, house officer], admission information (full name, date and time of admission, admission policy), vital signs, follow-up, discharge report approved by a consultant, and information concerning operation details (preoperative, intraoperative, postoperative), along with medical consent and consultations, as shown in Table 1.

**Table 1:** The new local standard

Standard	Description
1	Patient full name
2	Admission policy
3	Patient admission date
4	Patient admission time
5	Patient admission procedures
6	Vital signs documentation
7	Medical history
8	Physical examination
9	Documentation of investigations requested
10	Documentation of investigation results
11	Diagnosis of the diseases
12	Treatment plan or description
13	Approval of the treatment plan by the specialist
14	Documentation and completion of the implementation of the treatment
15	Complete medication instructions
16	Meaningful follow-up
17	Documentation of IV fluid chart and intake, output chart
18	Documentation of medical consultations
19	Documentation conversions or referrals between units
20	Medical consent
21	Description of pre-operative requirements
22	Accurate intra-operative description
23	Documentation of the recovery from postoperative
24	Patient discharge summary
25	discharge decision approved by the specialist
26	Time, date, and name in front of each document

## Results

In this study, 518 medical records (long-stay files) were evaluated using a checklist created by the Ribat Center for Quality and Excellence based on the data presented in the file (Table 2).

**Table 2:** Detailed evaluation of documentation completeness across 26 parameters in long-stay patient files in the general surgery department This table presents a comprehensive assessment of the documentation completeness, incompleteness, and absence for 26 key parameters in long-stay patient records. The parameters evaluated include patient demographics, medical history, diagnostic information, treatment plans, and discharge details. Each parameter's fulfillment is expressed as a percentage, reflecting the level of adherence to documentation standards in the General Surgery Department.

Standard	Description	Total N	Complete/fulfilled (%)	Incomplete (%)	Absent (%)
1	Patient full name	518	91 (17.6%)	426 (82.2%)	1 (0.2%)
2	Admission policy	518	108 (21%)	410 (79%)	0 (0%)
3	Patient admission date	518	447 (86.3%)	48 (9.2%)	23 (4.5%)
4	Patient admission time	518	10 (2%)	0 (0%)	508 (98%)
5	Patient admission procedures	518	30 (5.8%)	464 (89.6%)	24 (4.6%)
6	Vital signs documentation	518	71 (13.7%)	223 (43%)	224 (43.3%)
7	Medical history	518	422 (81.5%)	73 (14.1%)	23 (4.4%)
8	Physical examination	518	206 (39.7%)	193 (37.3%)	119 (23%)
9	Documentation of investigations requested	518	75 (14.5%)	22 (4.2%)	421 (81.3%)
10	Documentation of investigation results	518	365 (70.5%)	24 (4.6%)	129 (24.9%)
11	Diagnosis of the diseases	518	451 (87%)	25 (4.8%)	42 (8.2%)
12	Treatment plan or description	518	141 (27.2%)	32 (6.2%)	345 (66.6%)
13	Approval of the treatment plan by the specialist	518	10 (2%)	0 (0%)	508 (98%)
14	Documentation and completion of the implementation of the treatment	518	257 (49.6%)	94 (18.1%)	167 (32.3%)
15	Complete medication instructions	518	383 (74%)	67 (13%)	68 (13%)
16	Meaningful follow-up	518	334 (64.4%)	65 (12.6%)	119 (23%)
17	Documentation of IV fluid chart and intake, output chart	87	13 (14.94%)	14 (16.09%)	60 (68.97%)
18	Documentation of medical consultations	58	42 (72.4%)	0 (0%)	16 (27.6%)
19	Documentation conversions or referral between units	25	15 (60%)	0 (0%)	10 (40%)
20	Medical consent	415	298 (71.8%)	60 (14.5%)	57 (13.7%)
21	Description of pre-operative requirements	415	52 (12.5%)	7 (1.7%)	356 (85.8%)
22	Accurate intra-operative description	514	306 (73.7%)	61 (14.7%)	48 (11.6%)
23	Documentation of the recovery from postoperative	514	166 (40%)	0 (0%)	249 (60%)
24	Patient discharge summary	518	23 (4.4%)	10 (2%)	485 (93.6%)
25	discharge decision approved by the specialist	518	7 (1.4%)	0 (0%)	511 (98.6%)
26	Time , date, and name in front of each document	518	17 (3.3%)	402 (77.6%)	99 (19.1%)

The checklist contained 26 questions, and each question's documentation of fulfillment, incompleteness, or absence is evaluated. Not every patient in surgical departments needs surgery; some require non-surgical care and others require particular actions. Over 518 extended stay files, we discovered that 415 files had undergone surgery, 87 needed documentation for IV fluid charts (intake and output chart), 58 needed medical advice, and 25 needed communication or referral between units.

The results painted a varied picture. Some documents stood out with exceptional detail, capturing everything with precision, while others fell short, leaving critical gaps. The documentation of personal and disease information was incomplete, neither fully comprehensive nor entirely lacking, reflecting a mix of diligence and oversight. Fulfilled documentation for each result showed as follows: patient full name (17.6%), patient admission policy (21%), patient admission date (86.3%), patient admission time (2%), patient admission procedure (5.8%), vital signs documentation (13.7%), medical history (81.5%), physical examination (39.7%), documentation of investigation requested (14.5%), documentation of investigations results (70.5%), diagnosis of the disease (87%) treatment plan or description (27.2%), approval of the treatment plan by the specialist (2%), documentation and completion of the implementation of the treatment (49.6%), complete medication instruction (74%), meaningful follow-up (64.4%), documentation of IV fluid chart (14.9%), documentation of medical consultation (72.4%), documentation of referral between units (60%), medical consent (71.8%), pre-oprative requirements (12.5%), documentation of intraoperative description (73,7%), documentation of postoperative description (40%), patient discharge summary (4.4%), discharge descion approved by specialist (1.4%) and time and date, and name in front of each document (3.3%).

To assess the variation in documentation quality across different standards, we applied a chi-square test to compare the observed frequencies of complete, incomplete, and absent documentation in each category with the expected frequencies if documentation quality was uniform across all standards. The observed frequencies were taken from the audit results, which showed how many records met, partially met, or failed to meet the documentation requirements. By comparing these observed values with what would be expected if there were no differences between the standards, the test revealed a highly significant result (p < 0.001). This indicates that the completeness of documentation is not evenly distributed across the different standards, with some areas, such as patient admission times and treatment plan approvals, showing significantly poorer documentation than others, such as admission dates and diagnoses. This finding underscores the need for targeted improvements in specific areas to enhance overall documentation practices.

## Discussion

Patients' dates (86.3%), medical histories (81.5%), and diagnoses (87%) all had excellent documentation, indicating that the documenters - the majority of whom are interns - and perhaps this is due to the time that interns spend as medical students, during which time they pick up communication skills and the processes for reaching an accurate diagnosis. In their study on documentation, Piri et al. found that, while their percentage of 81% was similar to that of our study while recording histories [13], also 80.7% of medical history documentation and 79.9% of medical diagnoses in training hospitals in Iran showed the same close results [14]. The Imam Khomeini Hospital in Tabriz had a lower percentage in its records. They explain this due to inadequate attention to appropriate training of the students [13].

In contrast, time documentation revealed just 2% due to a decline in knowledge of the value of time. Comparing two secondary healthcare facilities in Egypt found that Kafr El-Sheikh General Hospital had poor documentation (16.5%), while El-Obor Health Insurance Hospital (84.5%) and El-Mahalla El-Qubra General Hospital (85.5%) had complete documentation. The study suggested that medical staff at all levels receive training on the value of proper documentation in order to address documentation deficiencies [15].

Documentation in the form of investigation results, implementation and medical instruction, follow-up, consultations, and referrals has been completed to an average level, and there are a number of reasons why. For example, most interns are satisfied with the paper results that are already in the file and place them there, which poses a major risk of loss. Documentation prevents this from happening, and this is due to a lack of awareness. On the other hand, investigations that were requested showed very poor results. Additionally, follow-up was lacking due to a lack of understanding of how to do it correctly, causing the low documentation in treatment implementation, plan and instruction, consultation, and referral, which are sometimes done verbally.

In terms of vital signs, 13.7% completed and 43% incomplete documentation is not up to par for a crucial record used to track patients' progress or decline. A comparison of documentation studies conducted in Egypt [16] and Basrah General Hospital [17] in family health (83.5%) and general surgery (18%), respectively, demonstrated wide variations between facilities. Along with the aforementioned factors, a lack of or halted equipment may also contribute.

Additionally, a reason for poor documentation is that some interns use papers rather than files of documentation because they sometimes neglect to notice the existence of different file sections, which prevents them from recording the information in their own section. Documentation of the IV fluid chart and intake-output chart was conducted in 87 medical record files; 68.9% were absent, and only 14.9% were completed. Better outcomes were seen in Chitwan Medical College Teaching Hospital, in which 50% were documented in the surgery department; however, the documentation is still weak, and they explained their deficiencies that medical records' poorly designed documentation makes them challenging to maintain [15]. Furthermore, the discharge summary, one of the poorest documented documents, showed only 4.4% completed for the same reason, and similar results were found in Nigerian tertiary hospitals (12.8%) [17].

The specialist's verbal permission of treatment and discharge is among the worst documentation because it involves their business. To make it better, we suggest doing some editing or delegating the task to other seniors if the specialist is too busy to do this task.

According to operational data collected from 415 out of 518 files, medical consent was shown to be 71.8% completed, while 13.7% were absent. Due to medico-legal considerations, this percentage should have been much lower. A study conducted at Jordan Hospital University between 2016 and 2021 to evaluate surgical records revealed an increase in the overall score. Of particular note was the consent score, which improved significantly from 8.5% in 2016 to 93.8% in 2021 after the required items were added. This supports the significance of ongoing auditing and appropriate intervention [18]. Preoperative requirements (85.5%) and postoperative (60%) absences of documentation were reduced for consideration by highlighting the significance of documentation. In the intraoperative description, the percentage needed to be greater than it can be (73.7%), and practice and discussion sessions will help with the improvements. Moreover, the review of the operation sheet for the surgical department at the Basrah General Hospital revealed poor documentation (76.6%), and they recommend a quality improvement project to improve the completion of medical records documentation [19].

Limitations

During the assessment of documentation for the medical audit, we faced a few challenges. One major issue was the disorganization of hospital files in the archive, which made it difficult to isolate and access the general surgery records. This disarray significantly increased the time required to identify and extract relevant files specifically for general surgery. Additionally, poor file preservation practices and inadequate storage conditions could lead to the failure or loss of records.

## Conclusions

Medical record documentation is essential for maintaining comprehensive patient information, supporting quality care, and meeting ethical and legal standards. A study in the General Surgery Department at Ribat University Hospital assessed the completeness of "long-stay" medical records. The findings revealed that, while some documents were well-maintained, the overall quality of documentation was inconsistent, with significant gaps in critical areas, such as patient admission times, specialist approvals, and discharge summaries. To address these deficiencies, increased supervision, enhanced training for medical trainees, regular audits, and the implementation of computerized systems are recommended to improve documentation practices.
